# Virtual Hand Illusion Induced by Visuomotor Correlations

**DOI:** 10.1371/journal.pone.0010381

**Published:** 2010-04-29

**Authors:** Maria V. Sanchez-Vives, Bernhard Spanlang, Antonio Frisoli, Massimo Bergamasco, Mel Slater

**Affiliations:** 1 Institució Catalana de Recerca i Estudis Avançats, Barcelona, Spain; 2 IDIBAPS, Barcelona, Spain; 3 Universitat Politècnica de Catalunya, Barcelona, Spain; 4 PERCRO, CEIICP, Scuola Superiore Sant'Anna, Pisa, Italy; 5 University of Barcelona, Barcelona, Spain; 6 University College London, London, United Kingdom; University of Regensburg, Germany

## Abstract

**Background:**

Our body schema gives the subjective impression of being highly stable. However, a number of easily-evoked illusions illustrate its remarkable malleability. In the rubber-hand illusion, illusory ownership of a rubber-hand is evoked by synchronous visual and tactile stimulation on a visible rubber arm and on the hidden real arm. Ownership is concurrent with a proprioceptive illusion of displacement of the arm position towards the fake arm. We have previously shown that this illusion of ownership plus the proprioceptive displacement also occurs towards a virtual 3D projection of an arm when the appropriate synchronous visuotactile stimulation is provided. Our objective here was to explore whether these illusions (ownership and proprioceptive displacement) can be induced by only synchronous visuomotor stimulation, in the absence of tactile stimulation.

**Methodology/Principal Findings:**

To achieve this we used a data-glove that uses sensors transmitting the positions of fingers to a virtually projected hand in the synchronous but not in the asynchronous condition. The illusion of ownership was measured by means of questionnaires. Questions related to ownership gave significantly larger values for the synchronous than for the asynchronous condition. Proprioceptive displacement provided an objective measure of the illusion and had a median value of 3.5 cm difference between the synchronous and asynchronous conditions. In addition, the correlation between the feeling of ownership of the virtual arm and the size of the drift was significant.

**Conclusions/Significance:**

We conclude that synchrony between visual and proprioceptive information along with motor activity is able to induce an illusion of ownership over a virtual arm. This has implications regarding the brain mechanisms underlying body ownership as well as the use of virtual bodies in therapies and rehabilitation.

## Introduction

The problem of self-recognition is concerned with how the central nervous system distinguishes what is part of the body and what is not. Although at first this might seem to be an easy problem to solve, for example, through different patterns of neural activity distinguishing between self-generated motor actions and the motor actions of others, research into mirror neurons shows that there are similar patterns of neural firing between watching an action performed by another and carrying out that action oneself. Jeannerod [1] discusses various possible contributors to self-recognition. One is the attribution of actions to the self (agency) through correlation between the intention to move and the resulting proprioceptive, and other multisensory signals and bodily responses. Another is the sense of ownership of the body caused by multisensory correlations between stimuli on the body, such as feeling a touch on a body part and at the same time seeing the visual correlate of the cause of the touch.

A demonstration that the problem of self-recognition is not straightforward is the fact that it is easy to generate illusions that involve misattribution of a rubber hand [2] or even a hand displayed in virtual reality [3] to the self. This is achieved through tactile stimulation of the hidden real hand and corresponding and synchronous visual stimulation on the visible fake hand. This rubber hand illusion involves not just subjective attribution of the rubber hand to the self, but also a mis-localization of where the stimulated hand is felt to be after a few minutes or even seconds of such synchronous visuotactile stimulation. When asked to blindly point towards the stimulated hand subjects will typically point towards the rubber or virtual hand – the distance between the real hand position and the indicated position being termed “proprioceptive drift”. Additionally, when the rubber hand is threatened, there are skin conductance responses indicating arousal, as if in preparation for pain [4]. When the visual-tactile stimulation is asynchronous, then the subjective, proprioceptive and arousal responses occur to a significantly lesser extent. For a review see [5].

Misattribution of an alien hand to the self as a result of motor actions rather than visual-tactile correlation has also been demonstrated. An experiment by Nielsen [6] showed that subjects will recognize the hand of an experimenter as their own, when their own hand is hidden and carrying out a drawing task that they see also being carried out by the experimenter's hand which is in a plausible position in relation to their own body. Moreover, when the experimenter's drawing deviates from the line that the subject is supposed to draw, the subjects tend to compensate for this, and yet remain unaware of the misattribution of the experimenter's hand as their own. Variations on this experiment [7], [8] showed that subjects tend to unconsciously and automatically follow visual cues in making corrections when the observed visual path of a stylus deviated from the path caused by their own motor actions, until the discrepancy became large enough that the conscious system took over in order to correct for bias. The point is that subjects would tolerate large mis-localization errors as well as misattribution in observations of the effects of their own motor actions.

There is also some evidence that an ownership illusion, akin to the rubber hand illusion, may be generated by synchronized visuomotor actions. Dummer et al. [9] carried out an experiment using a mechanical setup that moved a rubber hand synchronously or asynchronously with the hand movements of the subject, and compared each with a passive condition and the normal synchronous visuotactile rubber hand illusion. They found that the ownership illusion occurred with the visuomotor synchrony, although this was only demonstrated subjectively with a questionnaire. A note by Raz et al. [10] reports on an experiment using a hand projected in a stereo virtual environment (Reachin Display), where a questionnaire-based study found that a subjective illusion of ownership occurred both and separately for synchronous visuomotor and visuotactile stimulation.

In this paper we extend these results by exploiting a virtual reality system, and hand tracking with a data glove, showing that the illusion of ownership of the virtually presented hand occurs on the basis of visuomotor synchrony between movements of the real hand and the virtual hand. When there is asynchrony the illusion does not occur. This is demonstrated subjectively with a questionnaire, and behaviorally with proprioceptive drift, and additionally we observe significant positive correlations between proprioceptive drift and the questionnaire responses, akin to the original findings in [2].

## Materials and Methods

### Recruitment

Fourteen male participants with mean age 22.5±5.6 (S.D.) years were recruited for the experiment by advertisement on the university campus at Scuola Superiore Sant'Anna, Pisa, Italy. They were asked to read and sign an information consent form and they were paid 10€ for their participation. Participants were naïve with respect to the virtual/rubber hand illusion.

The work on body representation using virtual reality within the EU project PRESENCCIA has been approved by the ethics committee at the Hospital Clinic (Barcelona, Spain).

### Virtual Reality System

**Figure 1:**
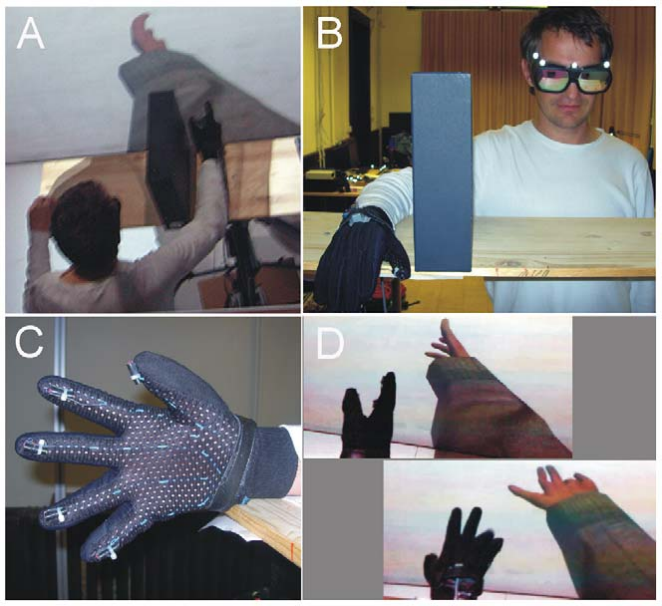
Experimental set up. **A**. The participant had his right arm resting on a tabletop. The arm was separated from view by a partition. The virtual arm was displayed on the screen in front of the participant. Its size and position was adjusted such that it looked correct from their point of view. The right hand was wearing the data glove. **B**. The participants (for the display one of the authors, BS, is represented) viewed from the front, wearing the stereo glasses and the data glove. **C**. Detail of the data glove. D. In the synchronous condition the virtual hand did follow the movements and finger position of the real hand tracked by the data glove.

The virtual reality set-up (Fig. 1A) consisted of a tracking system with a 6 degrees-of-freedom (DOF) Polhemus (http://www.polhemus.com/)Liberty head tracker (Fig. 1B) and a 2 m×2.7 m screen, where stereoscopic 3D images were back-projected by using an Infitec system (http://www.infitec.net/). The virtual environment was developed by using the XVR virtual reality platform (VRMedia http://www.vrmedia.it/).

We used a virtual character from the AXYZ design (http://www.axyz-design.com/) character set. The virtual character was visualised and animated in XVR by using a hardware accelerated library for character animation (HALCA) [11]. To the participant only the right arm and hand of the virtual character was shown. In HALCA the body mesh was deformed with the skeleton of the virtual character by using the dual quaternion skinning method [12] in a GPU vertex shader program.

Participants wore glasses with spectral filters (Infitec) for passive stereo viewing (Fig. 1B). In the synchronous condition their right hand rotations and displacements were tracked by a second 6DOF Polhemus Liberty tracker. The rotations were mapped to the forearm and hand rotations of a virtual character. The finger movements of the participant were tracked by the dataglove (Fig. 1C) described below and were also mapped to the finger bone joints of our virtual character's skeleton. In order to calibrate the finger movements we recorded the open and closed hand measured skeletal configurations of the finger bone angles for each subject before the experiment.

The program could log all the finger, hand and arm movements for later play back and analysis. In the asynchronous condition the arm, hand and finger movements of the virtual character were played back from a pre-recorded session.

A timer was programmed in the XVR scripting language to smoothly move for 20 s seconds the virtual character's arm to the left by a medial rotation around the shoulder 180 s after the start of the experiment. The timer was also used to play a beep sound to signal to the participant the end of the experiment and that the subject should point to where he thought his real hand was in order to enable us to measure the proprioceptive drift.

Subjects were fitted with a data glove worn on their right hand (Fig. 1C). This tracked the movements of their fingers, which drove a 3D virtual hand. The PERCRO data glove, developed by some of the authors at PERCRO, Scuola Superiore Sant'Anna, was used for the experiment. The data glove is equipped with patented absolute goniometric sensors [13] that can measure the angular displacements of proximal (MCP) and medial phalanxes (PIP) for all fingers and abduction-adduction of the thumb. The acquired angles are acquired on-line and used to reconstruct the full hand posture that is then mapped into a virtual 3-dimensional model of the hand and displayed to the user. Latency of the system was less than 5 msec.

### Experimental design and procedures

This was a repeat measures design. There was one between-group factor, which was the group to which subjects were assigned: Either they experienced the synchronous movement first followed by the asynchronous (group SA) or the other way around (group AS). There was one within groups factor – condition (synchronous or asynchronous):

1. Synchronous: The movements of the subject's own hand as captured by data glove determined the movements of the virtual hand (Fig. 1D).
2. Asynchronous: The virtual hand movements displayed were prerecorded and thus they were asynchronous with the movements of the real hand.

The experiment was carried out in a dark room, where the only light came from the screen. Volunteers stood with their right arm resting on a platform, occluded from their view by a partition (Fig. 1A). Their right hand wore the data glove (see above). The computer program generated a stereo image of a virtual arm (Fig. 1D; in the image in mono for better display). The virtual arm was positioned such that it seemed to be coming out the right shoulder, and it was also adjusted that it appeared to be the correct size. Unlike the real arm, the virtual arm was not shown resting on a shelf, but held outwards in front of the subject. The distance between the participant's real hand and the virtual hand was approximately 20 cm, the virtual hand being displaced towards the body.

Due to the head tracking, if the participant kept his body still and just moved his head as if to look at the arm from a different position, then the arm would appear to be stationary from a different point of view, as it would happen in reality. The setup was, therefore, able to powerfully induce the illusion that there was an arm pointing straight ahead, which appeared to be attached to the participant's body.

Once in the right position, subjects were told that whenever they heard a beep sound they should place one of two pieces of piece of blue-tack that they had been given and were holding in their left hand to a position under the board corresponding to where they felt the centre of their forearm to be.

The participants were then instructed to continually rotate their right hand along the prono-supination axis of their forearm and move their fingers as if they were counting, and this continued for 180 s. (The movement can seen in the video (Movie S1, S2) provided in the Supplementary Information). During this stage subjects were asked to concentrate their attention on the virtual hand in order to receive visual feedback of their movement in real time.

After this period of 180 s, the hand started drifting towards the left for 20 s, corresponding to a medial rotation of the right shoulder joint of 15 degrees with the elbow joint extended, covering a distance with the right hand of approximately 20 cm. At the end of this time there was another beep and the subject again placed a piece of blue-tack that they had been holding in their left hand under the board pointing towards where they felt position of the centre of their forearm to be.

### Questionnaire

After the experience, participants filled in an 11-item questionnaire (in Italian). Most questions were adapted and translated from [2] and some new questions were added. The labels are here added for convenience for the analysis of the results (Table 1). The questionnaire contained a set of assertions and was scored according to a 7-point Likert scale, where a score of 7 was described as ‘totally agree’ and a score of 1 as ‘totally disagree’ with the assertion.

**Table 1:** The Post-Experiment Questionnaire.

Variable Name	Assertion
**Ownership**	
*located*	I sometimes felt as if my hand was located where I saw the virtual hand to be.
*own*	Sometimes I felt that the virtual arm was my own arm.
**Illusion of movement**	
*affected*	I felt my own arm to be affected when I saw the virtual arm move to the left, at the end.
*influencing*	At some moments I felt that the movements of the virtual hand were influencing my own movements.
*drifted*	When the virtual arm drifted I felt that my real arm was drifting with it.
**Validity**	
*bythemselves*	The virtual hand and fingers seemed to be moving by themselves.
*caused*	The movements of the virtual hand and fingers were caused by my movements.
**Control**	
*morehand*	It sometimes seemed as if I might have more than one right hand or arm.
*between*	It sometimes seemed as if the position of the hand I was feeling came from somewhere between my own hand and the virtual hand.
*resemble*	The virtual hand began to resemble my own real hand, in terms of shape, skin tone, freckles or some other visual feature.
*virtual*	It sometimes felt as if my real hand was turning ‘virtual’.

The questionnaire statements were grouped into different types: two questions that indicated ownership illusion, which were designed to be as close as possible to those of [2] given the different experimental paradigm; three that referred to the illusion of movement; the two validity statements were chosen to check that the two experimental conditions operated as designed; and there were four control questions following the style of [2].

### Behavioral measure

In addition to the questionnaire, the proprioceptive drift elicited by the illusion was measured by a standard technique. Participants had been instructed to place the piece of blue-tack under the board were their forearm rested before and after the 200 s of experiment with eyes closed (see above). The position of the blue tack was immediately marked by an experimenter and then removed. The horizontal distance between both positions marked by the blue-tack corresponded to the proprioceptive drift.

## Results

### Questionnaire Results

First we compare the results on the synchronous (Movie S1) and asynchronous (Movie S2) conditions. We use a repeat measures one-way analysis of variance with between-groups variable Group (SA or AS) and one within-groups factor Condition (asynchronous or synchronous).

Table 2 shows the means, standard deviations, and the significance level for the difference between the means of Condition. There was no evidence of interaction between Group and Condition (in other words there was no order effect).

**Table 2:** Mean, SD, and Significance Levels for the Difference between Means, for the Asynchronous and Synchronous Conditions (Repeated Measures ANOVA) and the correlation (r) with proprioceptive drift in relation to Figure 3A and B.

	Asynch		Synch			Correlation	
Ownership	Mean	SD	Mean	SD	P	r	P
*located*	**2.8**	1.6	**4.1**	1.5	0.013	0.58	0.029
*own*	**2.9**	1.6	**4.9**	1.7	0.003	0.57	0.032
**Illusion of movement**							
*affected*	**3.6**	2.0	**3.6**	2.2	0.848	0.54	0.045
*influencing*	**4.4**	2.1	**3.6**	2.0	0.237	−0.42	0.133
*drifted*	**2.6**	1.6	**3.0**	1.8	0.449	0.36	0.203
**Validity**							
*bythemselves*	**5.9**	1.1	**2.1**	1.2	0.000	NA	NA
*caused*	**2.6**	1.5	**6.0**	1.4	0.000	NA	NA
**Control**							
*morehand*	**2.9**	1.6	**2.7**	1.8	0.709	−0.28	0.338
*between*	**2.5**	1.3	**3.4**	1.2	0.037	0.19	0.514
*resemble*	**3.4**	2.2	**4.3**	2.0	0.071	0.10	0.725
*virtual*	**2.9**	1.7	**5.0**	1.4	0.001	0.59	0.027

It can be seen that both the ‘illusion of feeling of ownership’ questions have significantly different means (higher for synchronous), however, none of the ‘illusion of movement’ questions have significantly different means. Amongst the control questions *between* and *virtual* were significantly different. Also there is some evidence of a difference for *resemble*. These three were also found to be significantly different between experimental and control group in a between-groups experiment using a virtual reality version of the rubber hand illusion.

The two consistency questions (*bythemselves* and *caused*) were appropriately significantly different.

Note that the residual errors of all models were tested for normality using the Jarque-Bera test, and the hypothesis of normality was never rejected (the smallest significance level was 0.33).

### Proprioceptive Drift

**Figure 2:**
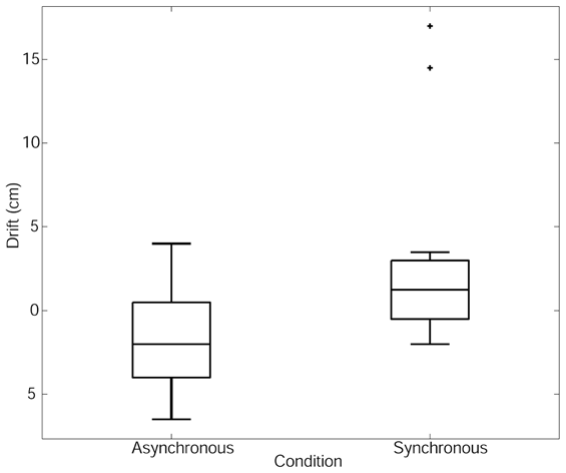
Standard Boxplots of the Drift for the Asynchronous and Synchronous Conditions.

Drifts were measured before and after each experimental trial as discussed above. The measurements (cm) were the horizontal distances, i.e., along a line parallel to the direction of virtual arm movement, the average drift being 3.25 cm. Figure 2 shows the drift by each condition. It suggests that the drift is higher for the synchronous condition, although there are two outliers in that condition. Since there was no reason to suspect either of these measurements, and if we removed them we would lose the balanced experimental design, we reduce their effect by replacing all drift measurements by their ranks, and carried out the repeated measures ANOVA (this is somewhat akin to the Kruskal-Wallis non-parametric ANOVA).

The ANOVA reveals that using the ranks (so that the absolute magnitudes of the outliers are not important) there is a significant difference between the two conditions as shown in Table 3, but that that there is no order effect (no difference between the groups). The Jarque-Bera test does not reject the hypothesis that the residual errors of the fit are normal (P = 0.85).

**Table 3:** Medians and Interquartile Ranges of Drift and Significance Level of Repeated Measures ANOVA on ranks of drift, for the test between the mean asynchronous and synchronous ranks.

	Asynchronous		Synchronous		
	Median	IQR	Median	IQR	P
Drift (cm)	**−2.0**	4.5	**1.25**	3.5	0.017

### Drift by Questionnaire Scores

**Figure 3:**
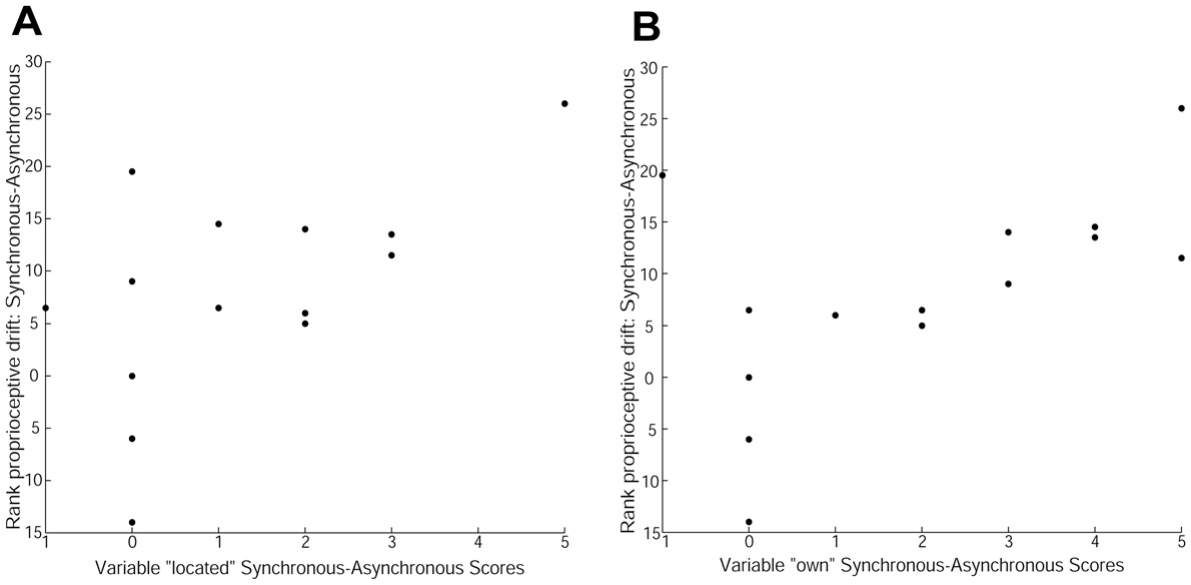
Scatter plots of questionnaire scores against rank drift. **A**. For the ownership variable “located”(Table 1). **B**. For the ownership variable “own” (Table 1).

In Figure 3A and B we show scatter plots of the rank drift against two of the questionnaire scores indicating ownership, “*located*” and “*own*” respectively (Tables 1 and 2). Specifically, the plots represent the differences between the questionnaire scores for the synchronous and asynchronous conditions, plotted against the differences in the rank drift between synchronous and asynchronous. Each of these ‘feeling of ownership’ questions shows a positive correlation.

Table 2 (last 2 columns) shows that there are significant positive correlations with the two questions indicating the feeling of ownership, and also with ‘virtual’.

## Discussion

The results we obtain with visuomotor synchrony are very similar to those of both the visuotactile synchrony based rubber hand [2] and virtual hand illusions [3]. In contrast to earlier experiments that have concentrated on movement synchrony in the rubber hand illusion [9], [10] we have also examined the behavioral proprioceptive drift measure and its correlation with the questionnaire scores. However, one difference in our experimental design compared with earlier ones was that during the period of the experiment (the first 180 s) the virtual hand was in a stable location, and then moved away from the real hand (the last 20 s). This did not lead either to a subjective illusion that the real hand was moving or to an actual movement of the real hand (as measured by the tracker on the glove). Nevertheless there was significant mis-localization of the hand when the subjects in the synchronous condition were asked to indicate their hand position at the termination of the virtual hand movement. In other words in spite of the fact that they saw the virtual hand move, did not feel their hand move, nor move it, they still blindly pointed towards the virtual hand when asked to point where they felt their hand to be.

This can be considered as a stronger result than the one obtained with the normal proprioceptive drift measure. In the latter, the rubber or virtual hand is seen to be stationary throughout the period of stimulation, so the conflict is between the stationary position of the real hand and the stationary fake hand. In these conditions it is known that there would be likely a significant misattribution of the real hand to another hand (for example, the hand of an experimenter) when either both are stationary or both are moving synchronously [14]. However, in our case the virtual hand was located approximately 20 cm away from the real hand for 180 s, and then it moved away. So to mis-localize the felt position of their hand, subjects had to negate not only the position of their real hand but also negate the fact that their real hand had not moved. Evidence for physiological changes during the RHI is given in [15] where it is shown that a drop in temperature can be observed in the hidden real hand. Although we have no physiological evidence in our experiment for this or for other physiological changes, our finding does suggest a substantial neglect of the real hand.

Makin et al. [5] presented a model for the RHI based on multisensory integration in peripersonal hand space which may be adaptable to the results presented here. In their model multisensory brain areas integrate the visual information of the fake hand with the proprioceptive information from the real hidden hand, but with the greatest weight given to the visual modality. Makin et al. mention the condition that the fake hand must be in a plausible position with respect to the body, which is satisfied in our experiment. They also suggest that the integration is weighted in favor of visual input provided that the real hand is static. In our case the real hand was not static but its movements either directly drove the movements of the virtual hand (synchronous condition) or the virtual hand made similar types of movements as the real hand but not the same movements (asynchronous condition). However, we could argue that in the synchronous condition the correlation between the visual movement and the proprioception would be enough to trigger the same recalibration of peripersonal space around the virtual hand as is the case for the RHI, so that the seen moving virtual hand triggers a unified visual-proprioceptive event centered on the virtual hand, with the real hand neglected. What is new here is that the unified visual-proprioceptive sensation is maintained even while the virtual hand moves its overall position away from the real hand (and note that the move was relatively slow - a 15 degree horizontal rotation about the shoulder lasting 20 s - and anatomically plausible). In fact it follows that if vision and proprioception become bound together, with vision dominating, then a move of the visual component should also result in a move of the proprioceptive component. Interestingly, this proprioceptive displacement was not conscious (as illustrated by the questionnaire responses) but only indicated by pointing towards the final observed hand position. It should also be noted here that it is unlikely that the proprioceptive drift occurred, for example, as a form of ‘suggestion’ – as a result of being induced by seeing the virtual hand move, or because the eyes of the subjects were caused to look in the direction of the moving hand with the pointing behavior following from this, since then we would have observed the same effect in the asynchronous condition. In a previous experiment [16], the proprioceptive illusion of displacement has also been induced by the realization of movement with one finger, while receiving visual feedback through the synchronously projected movement of the finger. Interestingly, the illusion of displacement occurs both with active and with passive movements of the finger, although it has different characteristics: when the movements that induce the illusion were active the perception of displacement was less than when passive, but the biases when pointing at a target were larger [16]. Here, we explored the proprioceptive displacement following active movements. However, following [16], we should have expected proportionally larger deviations had the subject been asked to point at targets. The maybe lesser degree of proprioceptive displacement that might have been expected after inducing ownership by active movements was in our case amplified by the artificial movement of the virtual hand. It should be noted that inducing ownership by visuomotor acts, or by agency, has been reported to have some different properties compared to inducing ownership by visuotactile correlations [2], [3], [17]. While visuotactile correlations of a finger may induce illusory displacements of individual fingers, visuomotor correlations of a finger movement induces an illusory drift of the arm, thus generating a more global, non-fragmented, body ownership [18].

Our final point is that virtual reality provides an excellent tool for studying body representation. The combination of stereo vision, tracking, haptic and auditory feedback, and the ability to represent even the body of the participant, generate an illusion for the participant of being and acting in an alternate virtual reality [19]. The evidence suggests that even the sense of what is their own body can be transferred to their virtual body representation. When the normal correlations between the different modalities are disrupted in a systematic way it is possible to produce the types of illusions that are important in understanding how the brain represents the body, especially when this can be combined with brain imaging.

## Supporting Information

Movie S1 Synchronous condition. Real hand in the data glove and virtual hand in the synchronous condition.
(3.43 MB MPG)

Movie S2 Asynchronous condition. Real hand in the data glove and virtual hand in the asynchronous condition.
(4.53 MB MPG)
